# Quad-Port Multiservice Diversity Antenna for Automotive Applications

**DOI:** 10.3390/s21248238

**Published:** 2021-12-09

**Authors:** Lekha Kannappan, Sandeep Kumar Palaniswamy, Lulu Wang, Malathi Kanagasabai, Sachin Kumar, Mohammed Gulam Nabi Alsath, Thipparaju Rama Rao

**Affiliations:** 1 Department of Electronics and Communication Engineering, SRM Institute of Science and Technology, Chennai 603203, India; lekhakannan13@gmail.com (L.K.); gupta.sachin0708@gmail.com (S.K.); ramaraotr@gmail.com (T.R.R.); 2 Biomedical Device Innovation Center, Shenzhen Technology University, Shenzhen 518118, China; 3 Department of Electronics and Communication Engineering, College of Engineering, Guindy, Anna University, Chennai 600025, India; mala@annauniv.edu; 4 Department of Electronics and Communication Engineering, SSN College of Engineering, Chennai 603110, India; alsath@live.com

**Keywords:** antenna, diversity, MIMO, monopole, vehicular communication

## Abstract

A quad-element multiple-input-multiple-output (MIMO) antenna with ultra-wideband (UWB) performance is presented in this paper. The MIMO antenna consists of four orthogonally arranged microstrip line-fed hexagonal monopole radiators and a modified ground plane. In addition, E-shaped and G-shaped stubs are added to the radiator to achieve additional resonances at 1.5 GHz and 2.45 GHz. The reliability of the antenna in the automotive environment is investigated, with housing effects taken into account. The housing effects show that the antenna performs consistently even in the presence of a large metal object. The proposed MIMO antenna has potential for various automotive applications, including vehicle-to-vehicle (V2V), vehicle-to-infrastructure (V2I), vehicle-to-everything (V2X), intelligent transport system (ITS), automatic vehicle identifier, and RFID-based electronic toll collection.

## 1. Introduction

The quest for high data rates has led to increased research into ultra-wideband (UWB) and multiband antennas. UWB antennas are used for a variety of automotive applications, including keyless entry, security, autonomous driving, vehicle-to-vehicle (V2V) communication, digital keys, and finding a vehicle in crowded parking lots. UWB technology can also be used for vehicle tracking, localization, and parking guidance. The vehicular antenna can be mounted in various locations, such as the back window, windshield, roof, or side mirror. The antenna may be readily fitted with the help of a shark-fin casing and chassis cavity.

Modern automobiles are becoming more intelligent, providing comfort and safety to drivers by enabling automated driving assistance and infotainment systems. Vehicle-to-everything (V2X) communication technology provides real-time traffic updates and safer driving by communicating directly with other vehicles. The key technologies for V2X communication in the unlicensed 5.9 GHz band are long-term evolution and wireless access in vehicular environments (WAVE). The notable features of V2X communication include automated driving with optimized fuel consumption, an integrated entertainment system, and the ability to send breakdown prevention information to drivers. In the literature, various antenna configurations have been proposed for automobile applications [1,2,3,4,5]. In [1], a square patch loaded with an inverted U-slot and a coupled C-slot was reported. In [2], a wheel-shaped fractal antenna was designed on the transparent polyvinyl chloride material substrate. In [3], a compact-sized dual-band antenna was presented with left-handed metamaterial. In [4], fractal geometry was used to design a microstrip patch antenna with a small footprint. In [5], a wheel-like fractal antenna was proposed for short-range communication, and the antenna was placed on a virtual car model to test its on-vehicle performance.

Signals in the automotive environment are received from multiple paths, resulting in multipath fading and interference. Diversity techniques such as spatial, pattern, and polarization are used to encounter multipath interference. Therefore, a multiple-input-multiple-output (MIMO)/diversity antenna could be advantageous for vehicular communications [6,7,8,9]. But, the main problem with MIMO antennas is high coupling between resonating elements. For improving inter-element coupling in uniplanar wideband MIMO antennas, electromagnetic band-gap (EBG) and other decoupling techniques were proposed in [10,11,12,13]. In [14,15,16,17], dual-port MIMO/diversity antennas with split-ring resonators (SRRs) were presented with improved isolation. In [18], a 3-D UWB MIMO configuration was reported, with the antenna elements positioned orthogonally to each other to reduce mutual coupling. An eight-element UWB MIMO antenna was designed [19], with the antenna elements placed orthogonally to offer dual polarization. A rectangular-shaped patch antenna was proposed in [20], with high inter-element isolation. A multiband dual-polarized antenna was developed in [21], where the antenna elements were placed orthogonally to increase isolation and achieve polarization diversity. An antenna working at multiple frequencies was presented in [22], where the resonating elements were arranged orthogonally to each other to reduce coupling between them. However, the antenna had a larger footprint. A Minkowski MIMO antenna was proposed in [23], where the Minkowski structure was obtained by cutting rectangular slots in the square patch, which helped increase isolation. In [24], a quad-port multiband MIMO antenna with pattern diversity and low inter-element coupling was reported. In [25], a quad-port antenna array was designed for 2G/3G/4G applications with isolation greater than 16.5 dB. In [26], a slotted microstrip antenna was proposed for WLAN applications, with a defected ground plane. In [27], an orthogonal orientation of elements was used to increase isolation in a quad-port triple-band antenna. In [28], a MIMO antenna was designed with meandering lines and SRR for improved isolation. However, the above-mentioned antennas are relatively large, have few resonating elements, and require more installation space.

This paper proposes a UWB MIMO antenna for automotive applications. The proposed antenna element has a straightforward geometry that covers a wide range of frequencies. Resonances at 1.5 GHz and 2.45 GHz are also achieved by incorporating stubs in the patch of the antenna element. The antenna elements are arranged orthogonally to develop the proposed MIMO antenna. The proposed quad-port MIMO antenna offers polarization and spatial diversity. The MIMO antenna is compact in size, covers multiple frequency bands, and offers good reliability in the automotive environment. It can be easily integrated into a vehicle using the shark fin mounting available in the market. The diversity parameters are also evaluated to understand the performance of the MIMO antenna, and the results are satisfactory.

## 2. Antenna Design

### 2.1. Antenna Element

**Figure 1:**
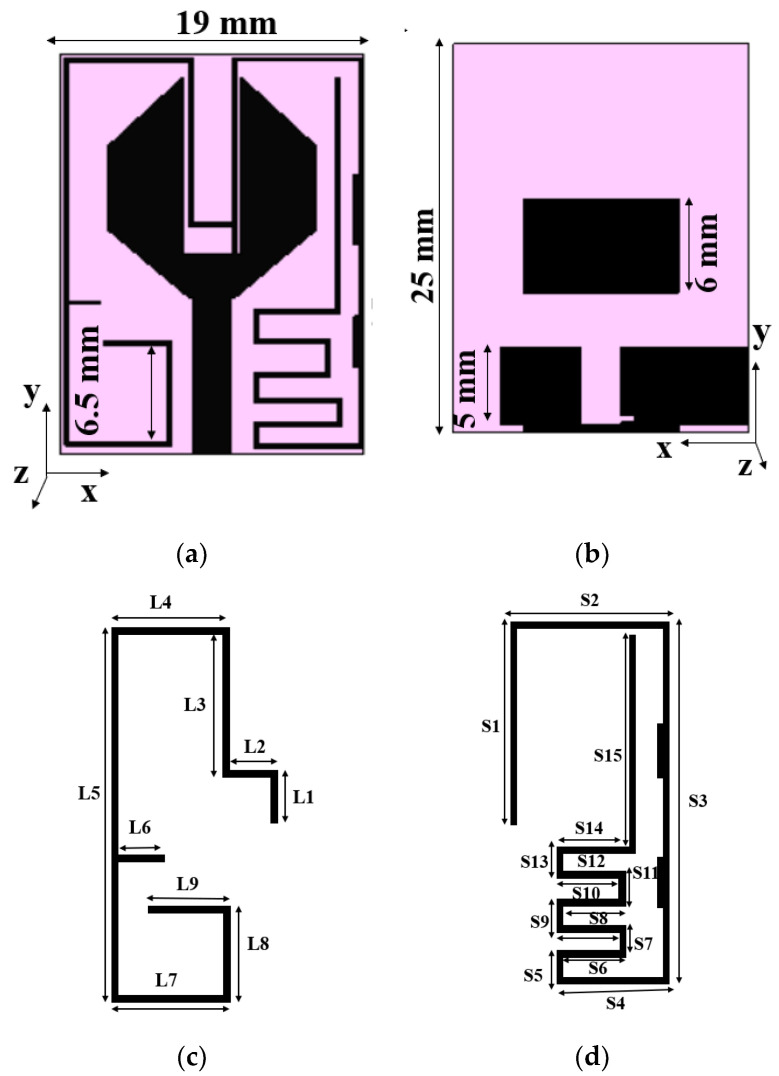
Layout of the antenna element: (**a**) front view, (**b**) back view, (**c**) G-shaped stub, 2.4 GHz, (**d**) E-shaped stub, 1.5 GHz.

Figure 1 shows the layout of the proposed antenna element. The antenna is developed on the FR-4 substrate with relative permittivity of 4.4 and a thickness of 1.6 mm. A simple hexagonal monopole radiator is combined with a 50 Ω feeding line and a modified ground plane to form the antenna element. The EM solver CST Microwave Studio^®^ is used to perform simulations of the proposed antenna. The size of the antenna element is 19 mm × 25 mm.

The lower band-edge frequency (*f_l_*) of the UWB monopole antenna is calculated as [29]

(1) $$f_{l}=\frac{7.2}{\left(l+r+p\right)\times k}$$

where *l* and *r* are the height and width of the antenna, respectively, and *p* is the distance between the ground plane and the radiator. The value of the empirical constant (*k*) is evaluated as

(2) $$k=\sqrt[{4}]{\varepsilon_{eff}}$$

For the proposed antenna, Equation (1) is modified as

(3) $$f_{l}=\frac{7.2}{\left(0.295\pi \left[\left(a+b\right)\right]+p\right)\times k}$$

where $0.295\pi \left[\left(a+b\right)\right]$ corresponds to the term (1+*r*), and *a* and *b* represent the semi-length and semi-width of the radiator, respectively.

Furthermore, resonant frequencies of 2.45 GHz and 1.5 GHz are obtained by adding G-shaped and E-shaped strips of length *λ*_0_/2, respectively. The wavelengths of G-shaped and E-shaped strips are calculated using the dimensions shown in Figure 1c,d. The dimensions of the G-shaped and E-shaped stubs are given in Table 1.

**Figure 2:**
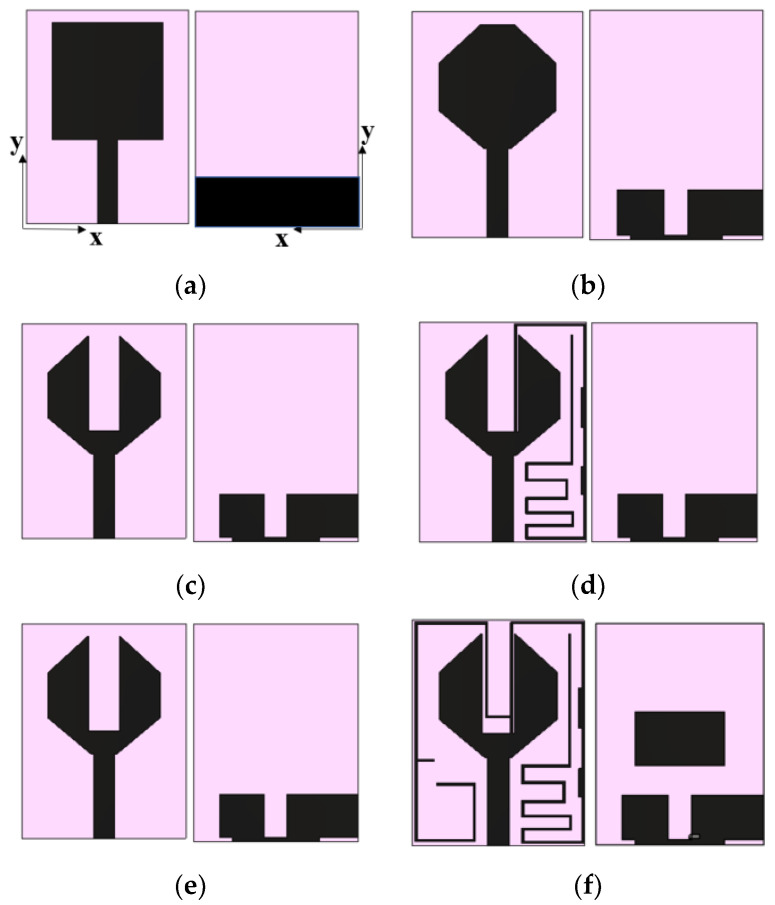
Evolution stages of the antenna element: (**a**) step 1, (**b**) step 2, (**c**) step 3, (**d**) step 4, (**e**) step 5, (**f**) step 6.

**Figure 3:**
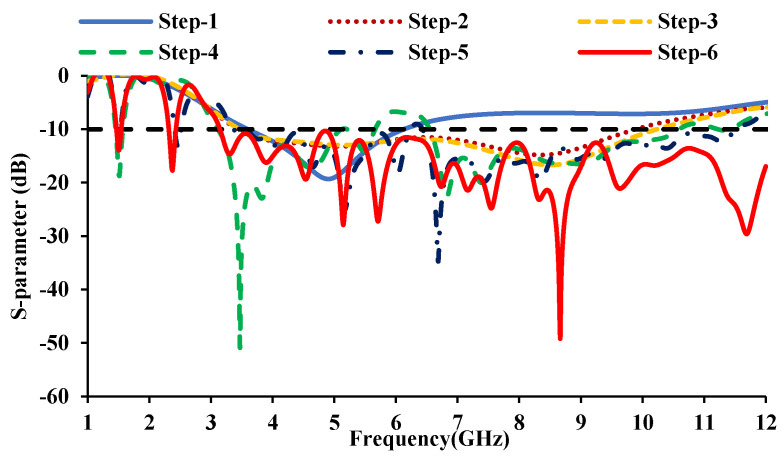
Reflection coefficients of the evolution steps.

**Figure 4:**
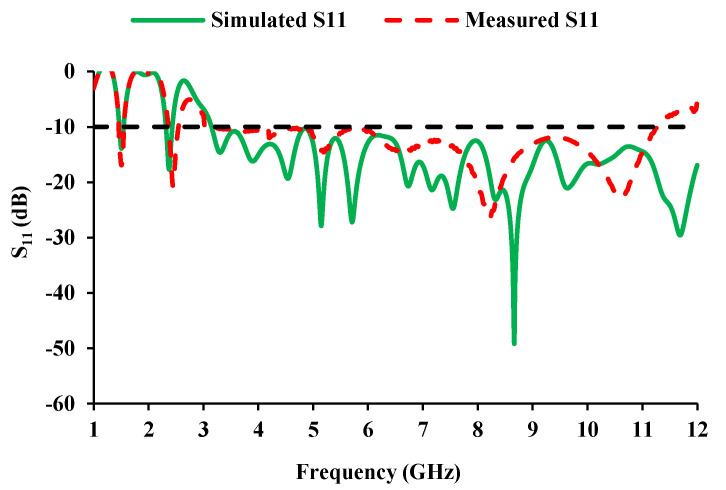
Reflection coefficients of the proposed antenna element.

The development stages of the proposed antenna element are shown in Figure 2. Figure 2a depicts a simple rectangular monopole antenna fed by a microstrip line of 50 Ω. The reflection coefficient characteristics of the evolution stages are shown in Figure 3. The rectangular monopole antenna exhibits poor impedance matching. In step 2, the edges of the radiator are truncated, and a defect in the ground plane is introduced for impedance matching. Step 3 involves etching the center metal of the radiator in order to reduce the physical size of the antenna. In step 4, an E-shaped strip is added to obtain resonance at 1.5 GHz, and a G-shaped strip is added (in step 5) to achieve additional resonance in the Bluetooth/Wi-Fi/RFID band (2.45 GHz). The addition of resonating stubs causes minor changes in impedance matching, which can be compensated by loading a patch in the ground plane, shown in step 6. Figure 4 shows the simulated and measured reflection coefficients of the proposed antenna element.

The antenna element offers an impedance bandwidth of 3.1 to 10.6 GHz and can be used for UWB automotive applications. The additional resonances (1.5 GHz and 2.45 GHz) can be employed for GPS and RFID/Bluetooth/Wi-Fi applications, respectively.

### 2.2. Equivalent Circuit

The antenna mechanism is studied physically by means of an equivalent circuit [30]. The equivalent circuit is derived from the impedance characteristics of the antenna. These resonances can be represented by the *R*, *L*, and *C* components. Two peak impedance points (5.124 GHz and 8.625 GHz) are chosen from Figure 4 (measured) and a corresponding circuit is derived.

**Figure 5:**
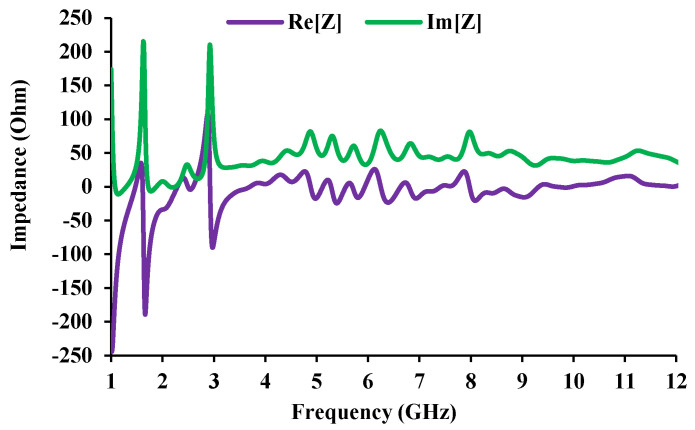
Impedance characteristics of the antenna element.

**Figure 6:**
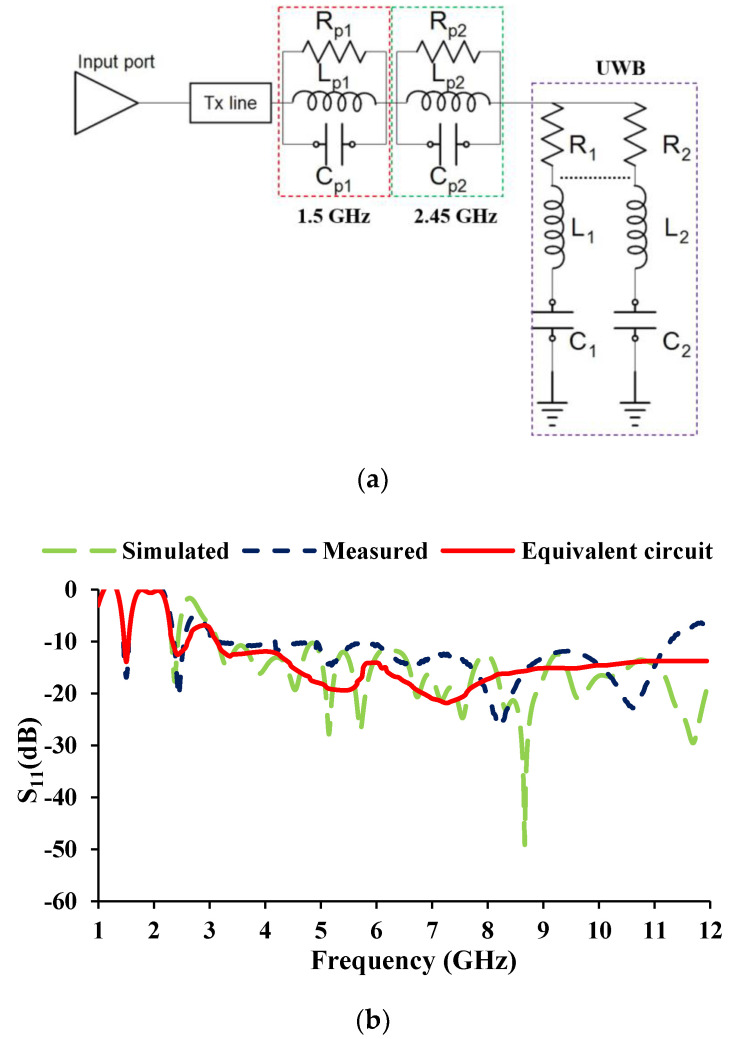
Equivalent circuit of the antenna (**a**) model, (**b**) S-parameters.

The impedance characteristics determine the type of resonant circuit. When the impedance moves from low (negative) to high (positive), a series resonance circuit is drawn, and when the impedance moves from high (positive) to low (negative), a parallel resonance circuit is drawn [31]. The real and imaginary curves (shown in Figure 5) can be used to draw the *RLC* equivalent of the antenna. The equivalent circuit of the antenna element and the corresponding result is shown in Figure 6, and the corresponding *RLC* parameters are given in Table 2. The 1.5 GHz and 2.45 GHz frequencies are contributed by two parallel resonant circuits, and the UWB is supported by two series resonant circuits.

### 2.3. MIMO Antenna

Multiple antennas are required in automobile applications to receive signals from all directions. The antenna elements in the proposed MIMO antenna are arranged orthogonally to one another to improve isolation, without the use of any decoupling structures. The orthogonal placement also provides polarization diversity.

**Figure 7:**
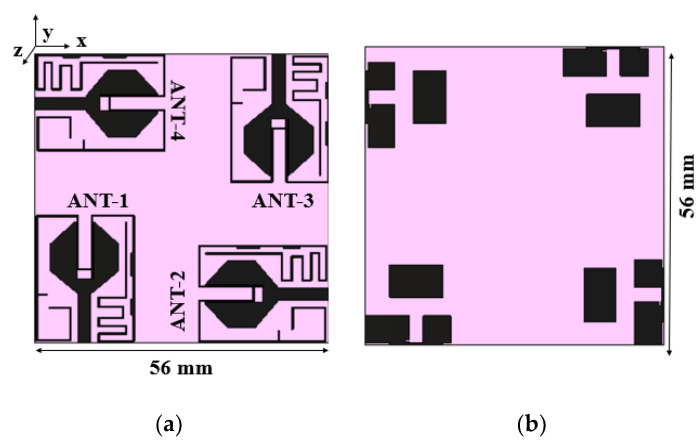
Layout of the MIMO antenna with unconnected ground planes: (**a**) front view, (**b**) rear view.

**Figure 8:**
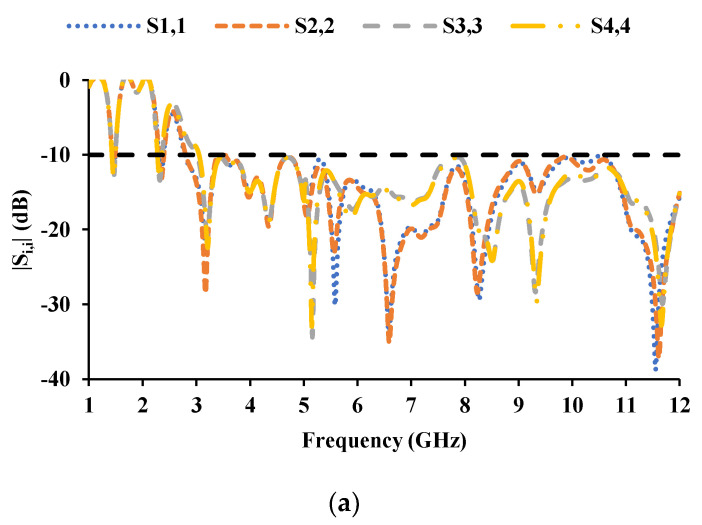

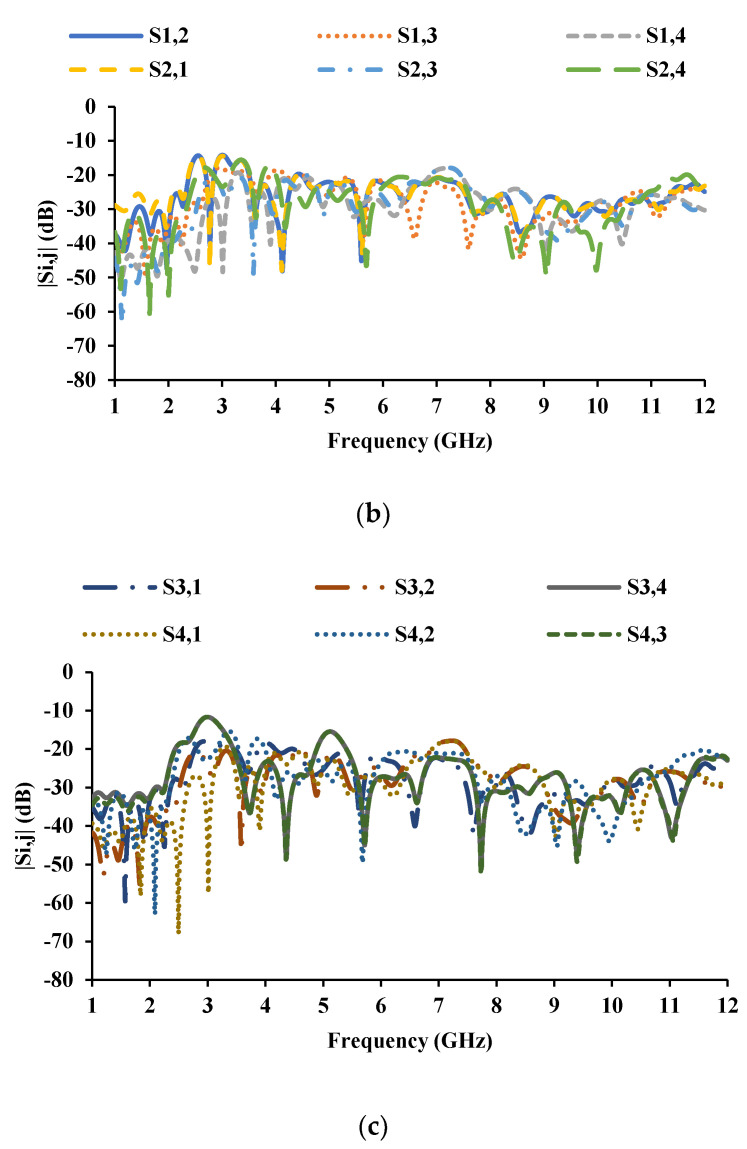
S-parameters of the MIMO antenna with unconnected ground planes: (**a**) reflection coefficients, (**b**) mutual coupling with respect to port 1 and port 2, (**c**) mutual coupling with respect to port 3 and port 4.

**Figure 9:**
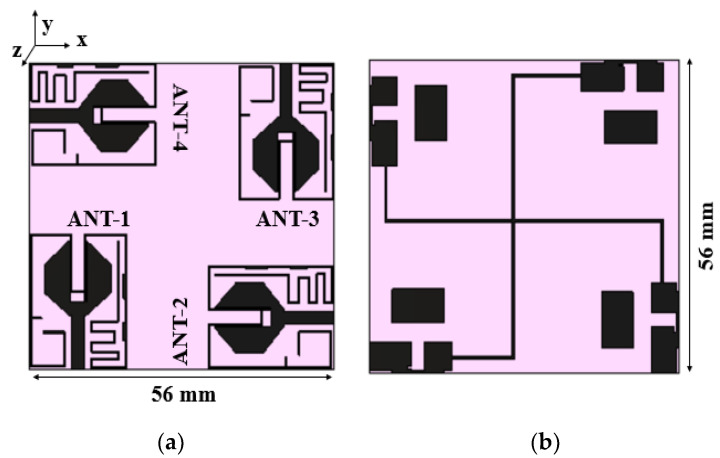
Layout of the proposed MIMO antenna with a common ground plane: (**a**) front view, (**b**) rear view.

**Figure 10:**
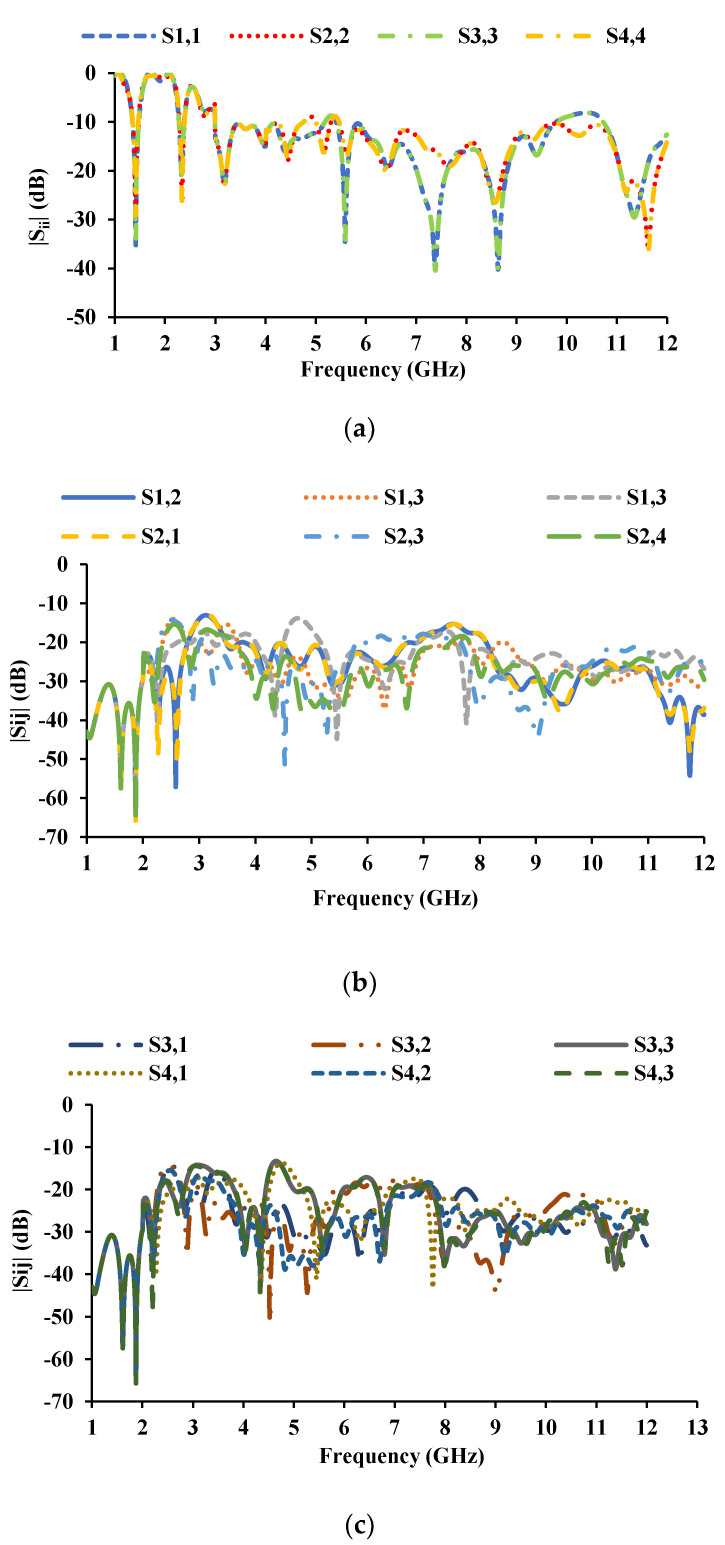
S-parameters of the MIMO antenna with the connected ground plane: (**a**) reflection coefficients, (**b**) mutual coupling with respect to port 1 and port 2, (**c**) mutual coupling with respect to port 3 and port 4.

**Figure 11:**
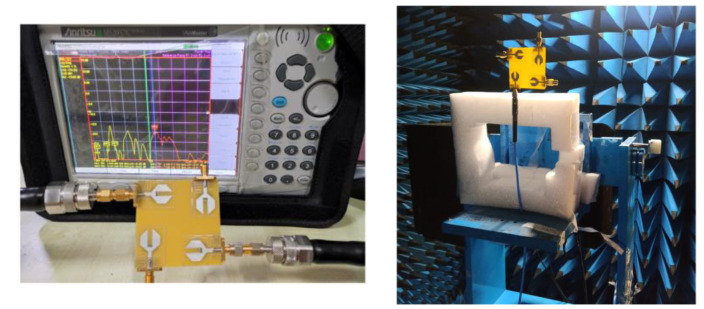
Proposed MIMO antenna prototype.

The inter-element spacing between the radiators is kept as 0.06*λ*_0_, and the MIMO antenna dimensions are 56 mm × 56 mm. Figure 7 and Figure 8 show the proposed MIMO/diversity antenna and its S-parameters without a connected ground plane, respectively. The MIMO antenna with a common ground plane [32] is shown in Figure 9. The reflection coefficients and mutual coupling of the MIMO antenna with a connected ground plane are presented in Figure 10a–c, respectively. There are no significant differences in the performance of the antenna with or without connected ground. The photograph of the proposed MIMO antenna prototype is shown in Figure 11.

## 3. Radiation and Diversity Characteristics

**Figure 12:**
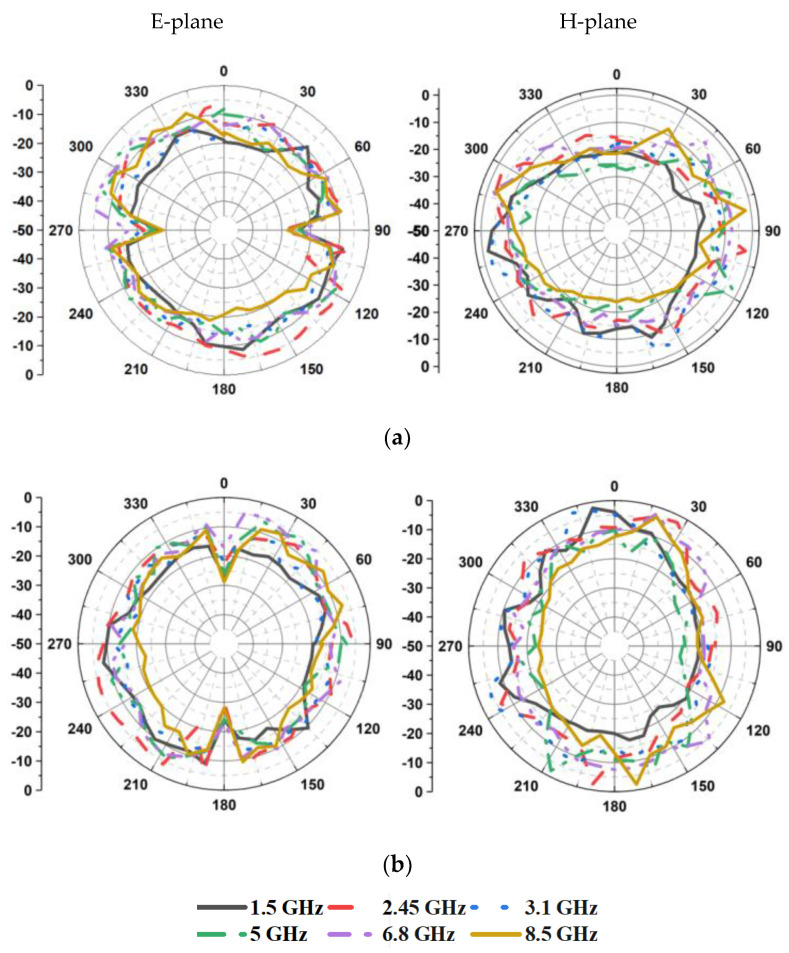
Measured radiation patterns of the antenna: (**a**) when port 1 is excited, (**b**) when port 2 is excited.

**Figure 13:**
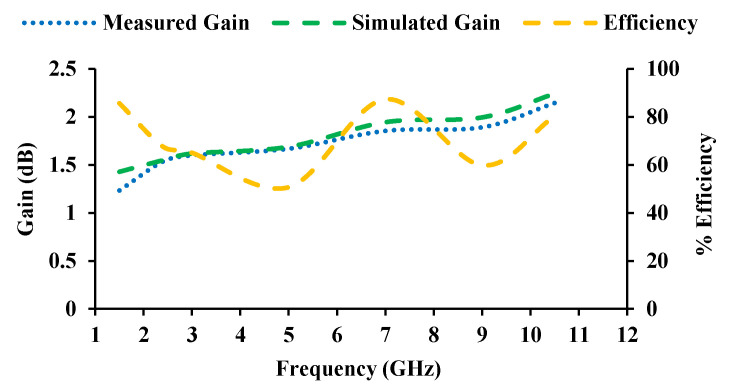
Measured gain and efficiency of the designed antenna.

The radiation patterns of the antenna in the E-plane and H-plane are displayed in Figure 12. Figure 13 depicts the gain and efficiency of the proposed antenna. The maximum gain is found to be 2.14 dBi, and the maximum efficiency is 87%.

**Figure 14:**
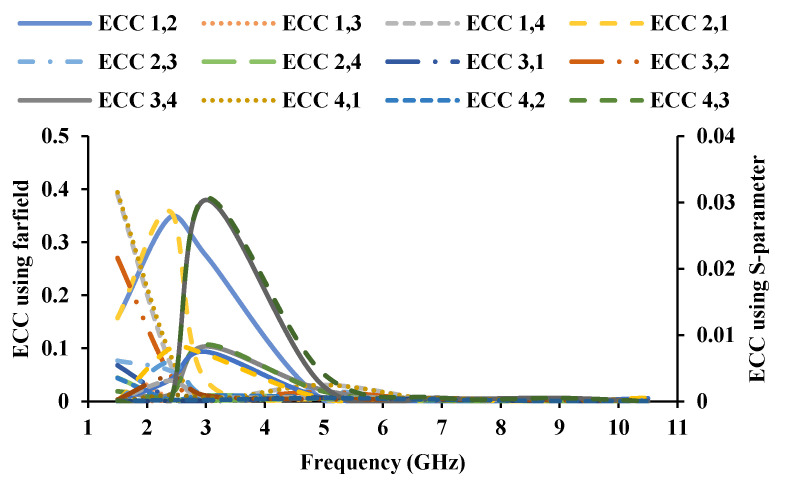
ECC of the MIMO antenna.

The diversity characteristics of the MIMO antenna are examined for its use in automobile applications. Envelope correlation coefficient (ECC) can be calculated with the S-parameter (Equation (1)) and the far-field (Equation (2)) [33], and the calculated ECC is less than 0.4, as shown in Figure 14.

(4) $$ECC\left(\rho_{e}\right)=\frac{{\left|S_{ii}^{*}S_{ij}+S_{ji}^{*}S_{jj}\right|}^{2}}{\left(1-{\left|S_{ii}\right|}^{2}-{\left|S_{ij}\right|}^{2}\right)\left(1-{\left|S_{ji}\right|}^{2}-{\left|S_{ii}\right|}^{2}\right)}$$

(5) $$ECC\left(\rho_{e}\right)=\frac{{\left|\iint \left[\vec{F_{1}}\left(\theta ,\varphi \right).\vec{F_{2}}\left(\theta ,\varphi \right)\right]dΩ\right|}^{2}}{\iint {\left|\vec{F_{1\;}}\left(\theta ,\varphi \right)\right|}^{2}dΩ\iint {\left|\vec{F_{2}}\left(\theta ,\varphi \right)\right|}^{2}dΩ}$$

**Figure 15:**
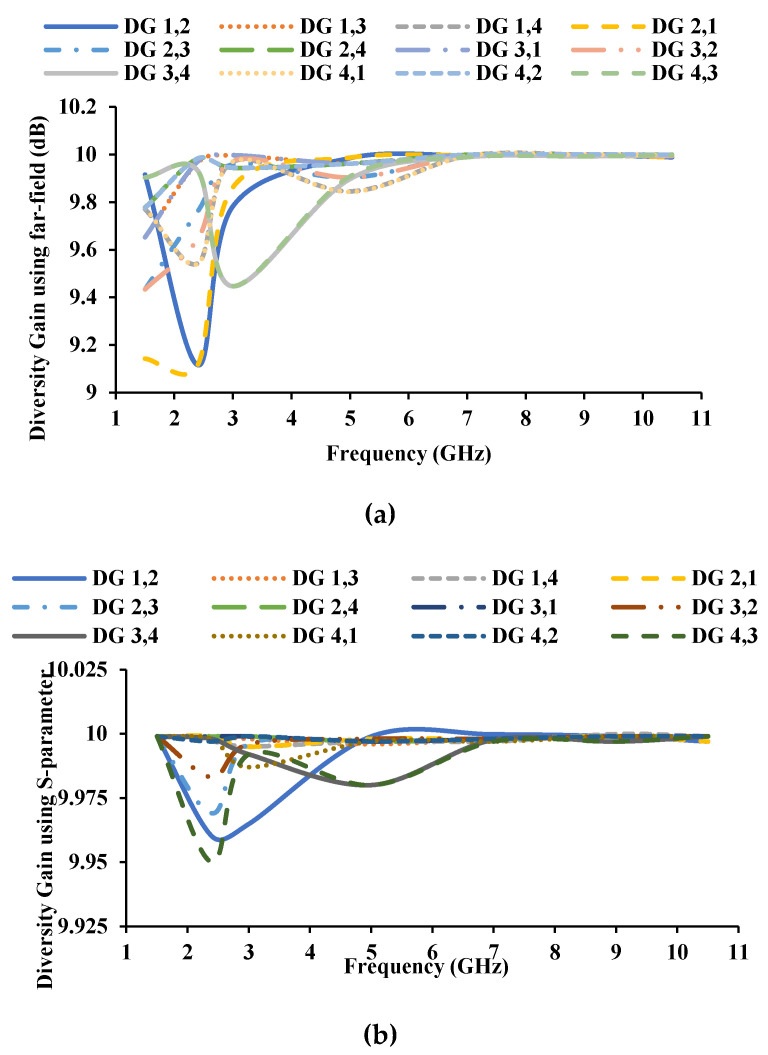
DG of the proposed antenna (**a**) using far-field, (**b**) using S-parameters.

Diversity gain (DG) is defined as an increase in signal-to-interference ratio without compromising quality [34]. It also demonstrates how much transmission power can be saved by employing a diversity scheme. It is measured in decibel or power ratio. DG can be calculated with the S-parameter and the far-field, and the DG of the antenna is shown in Figure 15.

(6) $$\mathrm{DG}=10\sqrt{1-{\left|\mathrm{ECC}\right|}^{2}}$$

**Figure 16:**
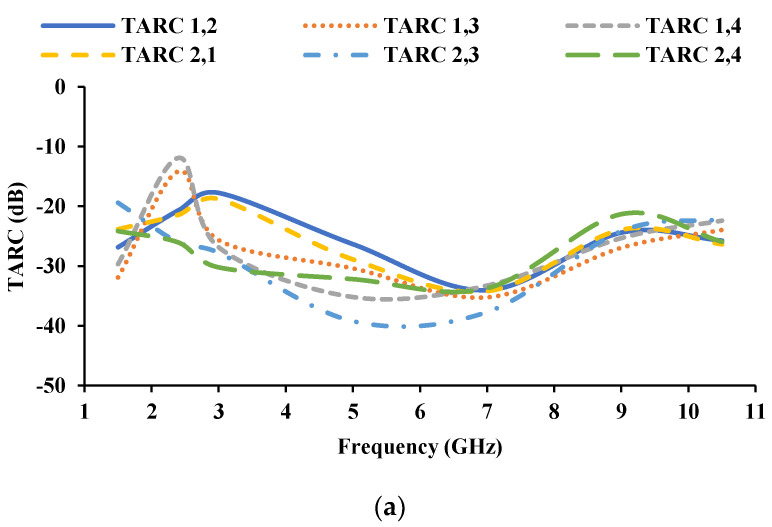

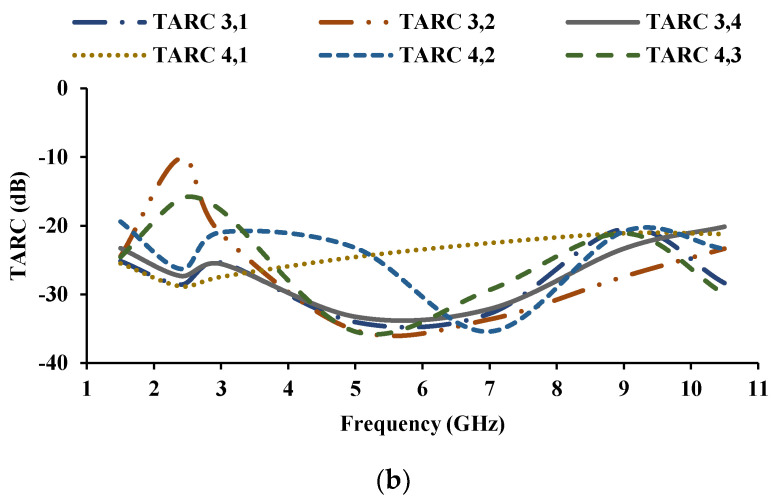
TARC of the proposed antenna (**a**) with respect to port 1 and port 2, (**b**) with respect to port 3 and port 4.

The total active reflection coefficient (TARC) is the square root of the summation of outgoing powers divided by the summation of incident powers at any port of an *N*-port antenna [35]. TARC is estimated using the Equation (7), where *a_i_* is the incident signal and *b_i_* is the received signal. TARC of the proposed antenna is less than −10 dB, shown in Figure 16.

(7) $$TARC=\frac{\sqrt{\sum_{i=1}^{N}{\left|b_{i}\right|}^{2}}}{\sqrt{\sum_{i=1}^{N}{\left|a_{i}\right|}^{2}}}$$

**Figure 17:**
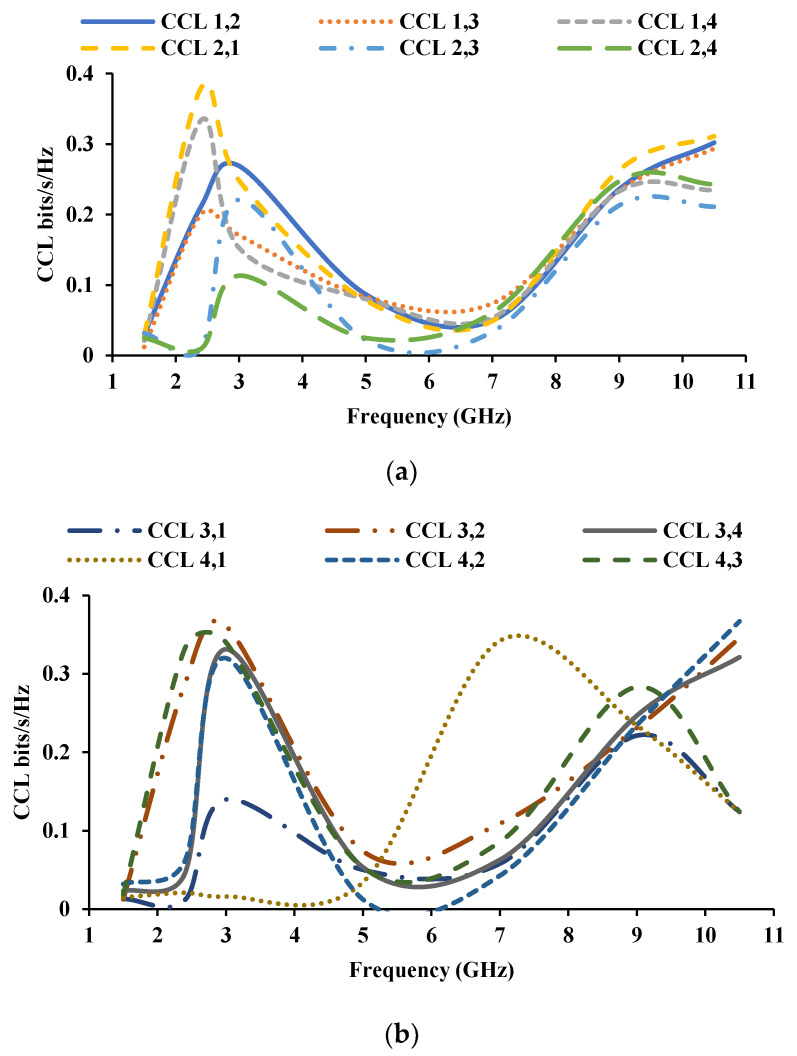
CCL of the proposed antenna (**a**) with respect to port 1 and port 2, (**b**) with respect to port 3 and port 4.

In high data rate transmission, the transmission loss is calculated using channel capacity loss (CCL) [36]. The CCL of the proposed antenna is shown in Figure 17, and it is less than the practical limit of 0.4 bits/s/Hz.

## 4. Housing Effect

**Figure 18:**
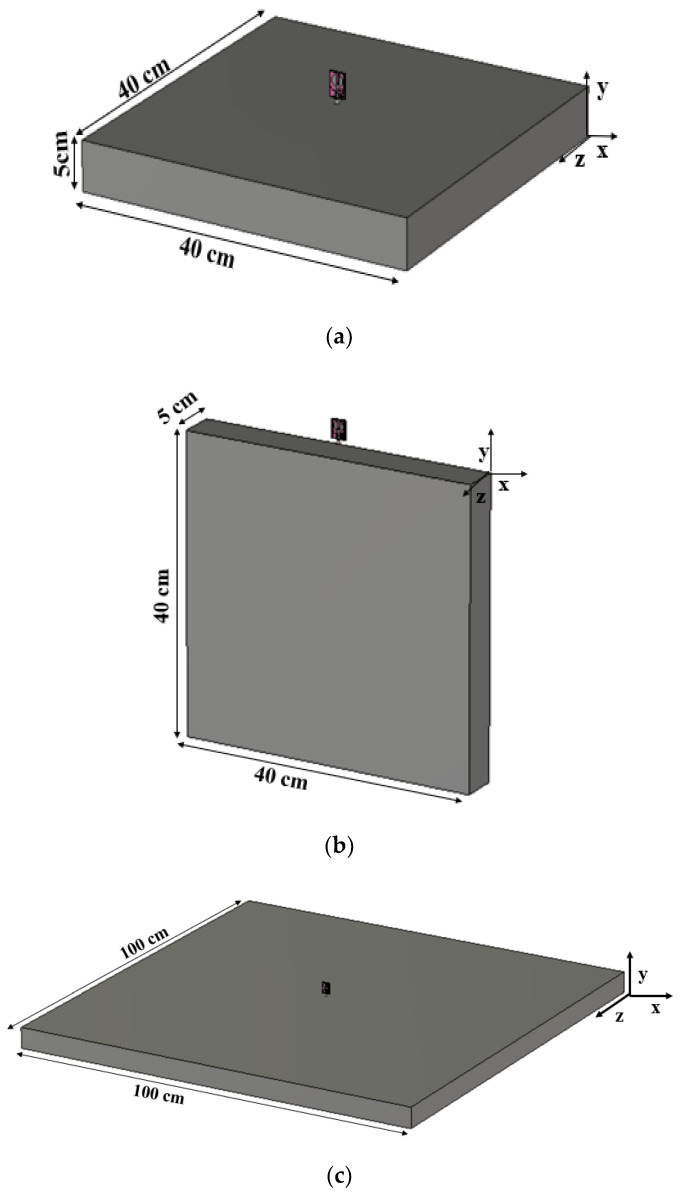

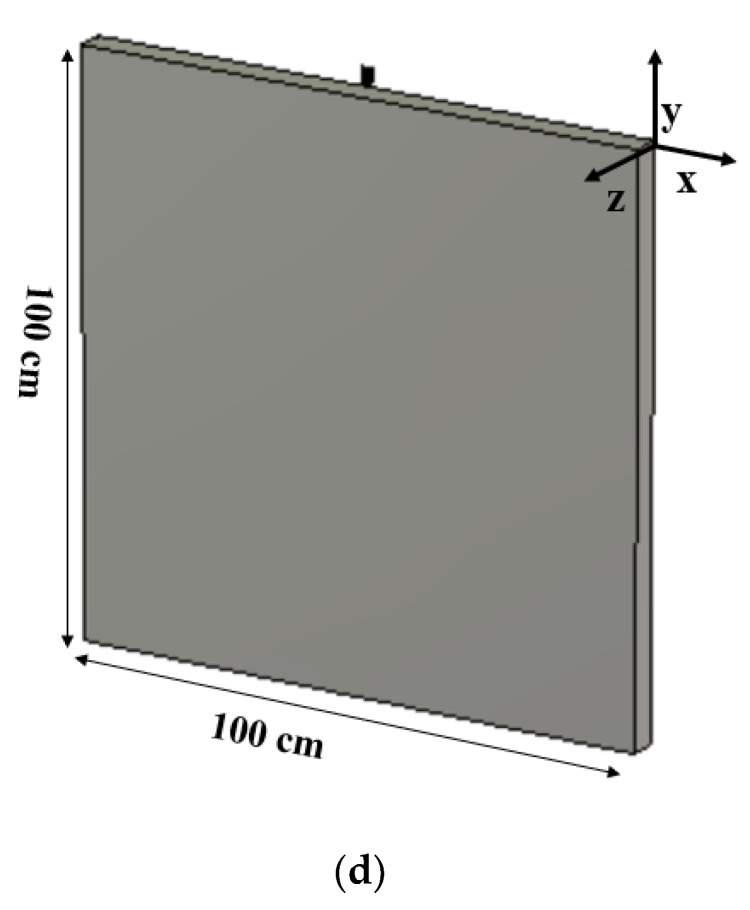
Housing effect of the antenna: (**a**) 40 cm × 40 cm, case-1, (**b**) 40 cm × 40 cm, case-2, (**c**) 1 m × 1 m, case-1, (d) 1 m × 1 m, case-2.

**Figure 19:**
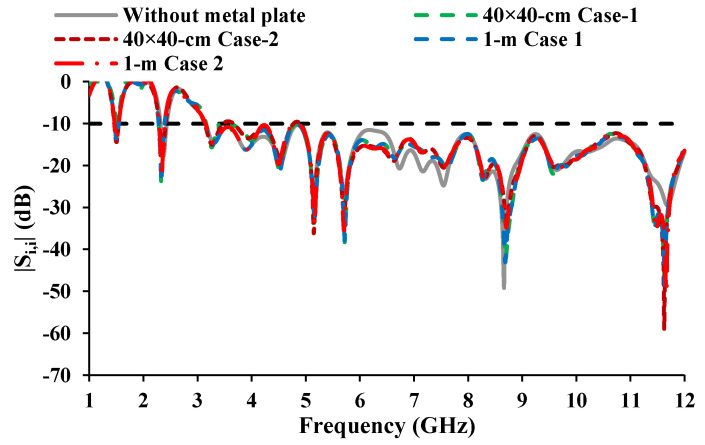
S-parameters of the antenna considering the housing effect.

The reliability of the antenna in the automotive environment is investigated, taking into account the housing effects [37]. The roof of the car is represented by a metal plate. The antenna is placed in the *xz*-plane and *yz*-plane, and the reflection coefficient characteristics in the presence of a conductor are investigated. The dimensions of the metal plate are kept as 40 cm × 40 cm and 1 m × 1 m (shown in Figure 18), and the gap between the antenna and the metal plate is marked as 10 mm. The results demonstrated (shown in Figure 19) that the performance of the antenna is not much affected by the metal conductor.

## 5. On-Car Scenario

**Figure 20:**
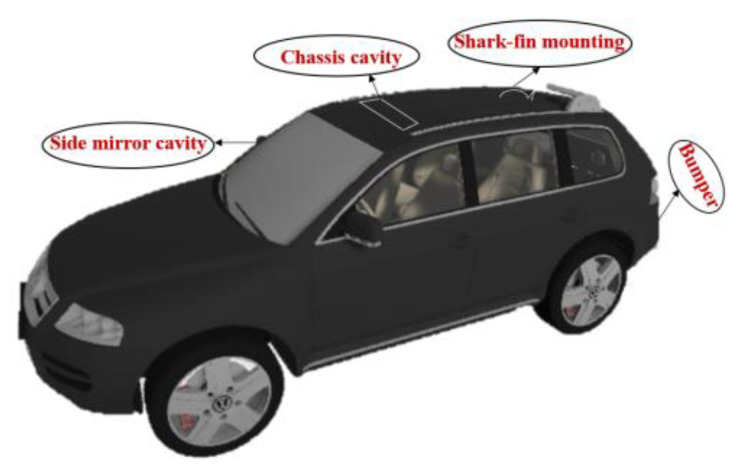
Different antenna mounting locations in a car.

The various locations in a vehicle where the proposed antenna can be installed are depicted in Figure 20. The antenna can be mounted on the bumper, roof, rear window, or side mirrors. The distance between the antenna and the ground should be larger to avoid ground losses. Therefore, the roof of the vehicle is the best place for antenna mounting. The antenna can be mounted on the roof using a shark-fin mounting system or in the chassis cavity.

**Figure 21:**
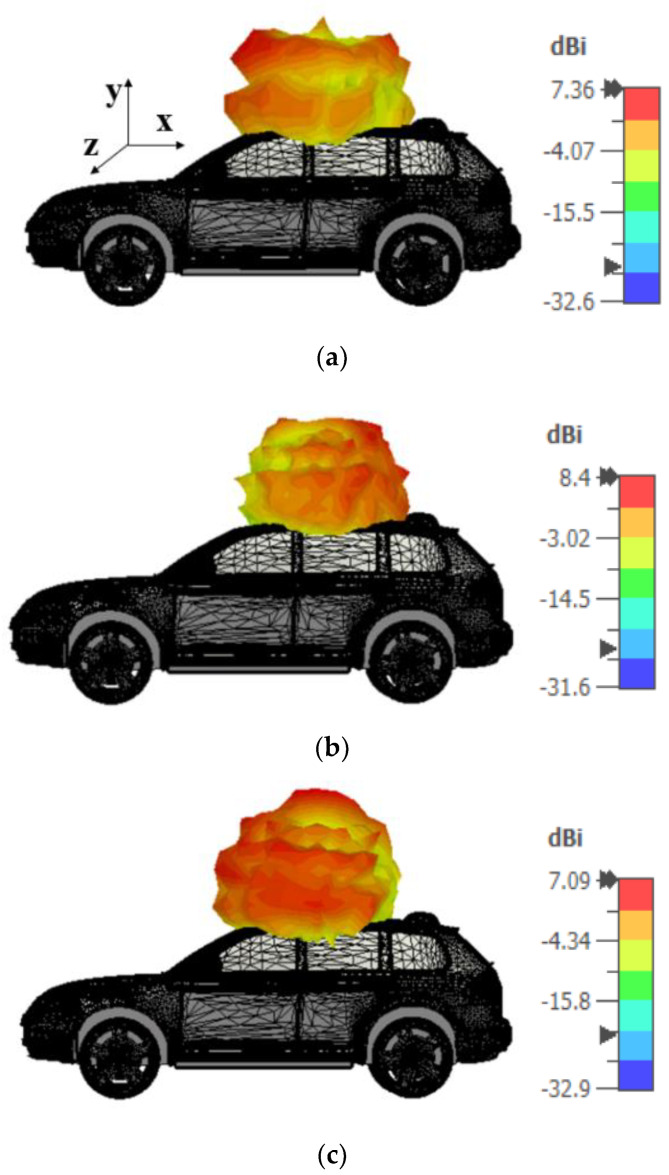
On-car performance of the proposed antenna: (**a**) 1.5 GHz, (**b**) 2.45 GHz, (**c**) 5 GHz.

Furthermore, the antenna is imported into an open-source CAD model to evaluate its directivity for on-vehicle conditions. Additionally, the far-field performance of the proposed antenna is investigated for an on-car scenario. The results showed that the antenna has omnidirectional characteristics over the desired frequencies. The proposed antenna has directivity greater than 7 dBi, as shown in Figure 21.

A comparison of the proposed antenna to previously reported antennas is shown in Table 3. The salient features of the proposed antenna are as follows:

1. The proposed antenna covers multiple bands, including UWB, whereas other antennas reported in the literature [38,39,40,41,42,43,44,45,46,47,48,49,50,51,52,53] operate on a single wideband frequency.
2. The proposed antenna requires less space than the antenna configurations reported in [39,40,41,42,44,45,46,47,48,49,50,51,52,53,54,55].
3. The multiband operation is achieved in the proposed prototype without any need for reconfigurability.
4. The orthogonal configuration of the antenna provides additional polarization and covers signals from all directions.
5. The MIMO antenna diversity performance is investigated using parameters such as ECC, DG, TARC, and CCL, and the calculated values are found in the acceptable limits.
6. The proposed antenna is tested in an automotive environment, and the results show that the performance of the proposed antenna is stable.

The proposed antenna has the potential for automotive applications such as V2V, vehicle-to-infrastructure (V2I), V2X, intelligent transport system, automatic vehicle identifier, and RFID-based electronic toll collection.

## 6. Conclusions

A MIMO/diversity antenna for automotive communications is designed and developed in this paper. Stubs are integrated into the UWB monopole antenna element to achieve resonance at 1.5 GHz and 2.45 GHz. The automotive antenna must receive signals from all directions; therefore, the antenna elements are arranged orthogonally to each other. The orthogonal arrangement of the antenna elements also improves inter-element isolation. The antenna is fabricated and tested for diversity performance, and the obtained results show that the ECC is less than 0.4, DG is greater than 9 dB, TARC is greater than −10 dB, and CCL is less than 0.4 bits/s/Hz. The proposed MIMO antenna could be helpful for GPS, RFID/Bluetooth/Wi-Fi, and V2V communications.

## Figures and Tables

**Table 1:** Dimensions of the G-shaped and E-shaped stubs.

Dimension	Value (mm)	Dimension	Value (mm)
*L*1	1.7	*S*4	6.9
*L*2	2.75	*S*5	1.7
*L*3	10.6	*S*6	5.5
*L*4	8.05	*S*7	1.7
*L*5	22.3	*S*8	5.5
*L*6	1.9	*S*9	1.85
*L*7	6.7	*S*10	4.8
*L*8	6.5	*S*11	2.35
*L*9	4.3	*S*12	4.8
*S*1	12.3	*S*13	2.1
*S*2	8.15	*S*14	5.4
*S*3	24.5	*S*15	14.75

**Table 2:** *RLC* parameters of the equivalent circuit.

Frequency (GHz)	*R* (Ω)	*C* (pF)	*L* (nH)
1.53	38.93	23.55	0.459
2.43	55.11	13.38	0.321
4.504	50.6	0.44	2.837
8.656	50.39	0.0739	4.571

**Table 3:** Comparison of the proposed antenna to previously reported antenna configurations.

Ref.	Bandwidth (GHz)	Dimensions (*λ*_0_ × *λ*_0_)	Substrate	Peak Gain (dBi)	Efficiency (%)	DG (dB)	ECC	Polarization
[38]	2–11	0.22 × 0.22	FR-4	3	75	10	<0.5	Dual
[39]	3.1–10.6	0.39 × 0.39	FR-4	5	---	10	<0.02	Dual
[40]	25.5–29.6	2.5 × 2.97	Rogers RO4350B	8.3	82	>9.96	<0.01	Dual
[41]	3.1–11	0.46 × 0.46	FR-4	5.5	---	>9.9	<0.015	Vertical
[42]	3.2–11	0.38 × 0.38	FR-4	4	>70	>9 (ADG)	<0.5	Dual
[43]	2.2–12.3	0.19 × 0.19	FR-4	5.82	87	>8 (ADG), >7.5 (EDG)	<0.3	Vertical
[44]	3.1–10.6	0.82 × 0.82	FR-4	3.38	>85.7	---	<0.001	Dual
[45]	3.1–11.9	0.37 × 0.37	FR-4	6	>78	>9.96	<0.03	Dual
[46]	3–11	0.39 × 0.3	Rogers 5880	5.8	-	>9.8	<0.02	Vertical
[47]	5.1–5.8	0.85 × 0.85	FR-4	2.9	>70	>9.9	<0.0006	Dual
[48]	2.35–9.04	0.51 × 0.27	FR-4	3	-	8	<0.5	Vertical
[49]	3–16	0.58 × 0.58	FR-4	7	-	-	<0.07	Dual
[50]	3.14–12.24	0.524 × 0.524	Rogers 3003	5.1	>81	>9.6	<0.004	Dual
[51]	3.8–6.5	0.57 × 0.57	FR-4	6.8	>60	-	<0.04	Circular
[52]	3–13.5	0.4 × 0.4	TMM4 laminate	3.5	>89	>9.95	<0.4	Dual
[53]	2.1–11.4	0.24 × 0.32	FR-4	1.2	>75	>9.9	<0.04	Dual
[54]	5.5–9.2, 13.2–17.9, 11.5–14.6	0.733 × 0.733	FR-4	7.57 dB	>70	>9.9	<0.05	Dual
[55]	2.15–20	0.308 × 0.308	FR-4	6.7	>60	>9.96	<0.01	Dual
This work	1.41–1.62, 2.4–2.462, 3.1–12.8	0.2 × 0.2	FR-4	2.14	87	>9	<0.4	Dual

## Data Availability

The data presented in this study are available on request from the corresponding author.
